# The role of Arabidopsis aldehyde dehydrogenase genes in response to high temperature and stress combinations

**DOI:** 10.1093/jxb/erx194

**Published:** 2017-07-11

**Authors:** Junyi Zhao, Tagnon D Missihoun, Dorothea Bartels

**Affiliations:** Institute of Molecular Physiology and Biotechnology of Plants (IMBIO), University of Bonn, Kirschallee, Bonn, Germany

**Keywords:** Acquired thermotolerance, aldehyde dehydrogenases, *Arabidopsis thaliana*, basal thermotolerance, high temperature stress, stress combinations

## Abstract

Aldehyde dehydrogenases (ALDH) are a family of enzymes that are involved in plant metabolism and contribute to aldehyde homeostasis to eliminate toxic aldehydes. The ALDH enzymes produce NADPH and NADH in their enzymatic reactions and thus contribute to balancing redox equivalents. Previous studies showed that Arabidopsis *ALDH* genes are expressed in response to high salinity, dehydration, oxidative stress, or heavy metals, suggesting important roles in environmental adaptation. However, the role of *ALDH* genes in high temperature and stress combinations (heat stress combined with dehydration, wounding, or salt stress) is unclear. Here, we analysed expression patterns of selected *ALDH* genes on the transcript and protein level at different time points of heat stress, basal and acquired thermotolerance, and stress combination treatments. Our results indicate that ALDH3I1 and ALDH7B4 are strongly induced by heat stress. Higher levels of ALDH7B4 accumulated in response to dehydration–heat, heat–salt and wounding–heat combination stress than in response to single stressors. The comparison of physiological and biological parameters in T-DNA double mutants of *ALDH* genes and wild-type plants demonstrated that mutant lines are more sensitive to heat stress and stress combinations than wild-type plants.

## Teaser

The expression of selected Arabidopsis *ALDH* genes is induced in response to heat and stress combination, and *ALDH* mutant lines are more sensitive to heat stress and stress combinations.

## Introduction

Current climate predictions indicate that average surface temperatures will rise by 3–5 °C during the next 50–100 years and crop plants will face an increase in weather disasters that can have severe consequences (Teixeira *et al.*, 2013). Temperature stress can have a devastating effect on plant metabolism, disrupting cellular homeostasis and uncoupling physiological processes (Wahid *et al.*, 2007). A direct result of stress-induced cellular changes is the enhanced accumulation of cellular toxic compounds including reactive oxygen species (ROS), which in turn lead to excessive aldehyde accumulation (Stiti *et al.*, 2011; Singh *et al.*, 2013). Aldehydes are intermediates in several fundamental metabolic pathways and they are produced in response to salinity, dehydration, desiccation, cold, and heat stress (Bartels, 2001; Kirch *et al.*, 2005; Kotchoni *et al.*, 2006). Aldehydes may react with proteins and nucleic acids and thus destroy their functions, which consequently leads to cell death. Excessive aldehydes can be removed by active ALDH enzymes.

The aldehyde dehydrogenase (ALDH) enzymes belong to a family of NAD(P)^+^-dependent enzymes that have substrate specificity and catalyse the oxidation of various aldehydes to the corresponding carboxylic acids, thus reducing the peroxidation of lipids. *ALDH* genes are considered to be ‘aldehyde scavengers’ to eliminate surplus aldehydes (Sunkar *et al.*, 2003; Kirch *et al.*, 2004). They are involved in stress adaptation to biotic and abiotic environments and regulate aldehyde homeostasis under stress conditions (Perozich *et al.*, 1999). Stress-related members of *ALDH* genes have been investigated in Arabidopsis (Stiti *et al.*, 2011). Previous studies demonstrated that constitutive or stress-inducible expression of both the chloroplastic *ALDH3I1* and the cytoplasmic *ALDH7B4* confer tolerance to osmotic and oxidative stress in transgenic plants (Kirch *et al.*, 2005). Stress tolerance in transgenic plants overexpressing *ALDH3F1*, *ALDH3I1* or *ALDH7B4* genes is accompanied by a reduction of H_2_O_2_ and malondialdehyde (MDA). Involvement of *ALDH* genes in stress tolerance was corroborated by the analysis of Arabidopsis *ALDH* T-DNA knock-out (KO) mutants. Mutant lines were more sensitive to dehydration and salt stress than wild-type plants (Sunkar *et al.*, 2003; Kotchoni *et al.*, 2006; Missihoun *et al.*, 2012).

Individual abiotic stresses are well studied, but much less is known about the effect of multiple, co-occurring stress factors, despite the fact that multiple stresses are probably the rule under natural conditions (Holopainen and Gershenzon, 2010). Recent research shows that the response of plants to a combination of two different abiotic stresses cannot be directly extrapolated from the response of plants to the individual stress (Mittler, 2006). For example, plants open their stomata to cool the leaves during heat stress, but if heat and dehydration occur simultaneously, the dehydration-induced stomatal closure will cause a reduction in stomatal conductance and higher leaf temperature (Rizhsky *et al.*, 2004). Likewise, when salinity or heavy metals occur in combination with heat stress, enhanced transpiration could result in the uptake of salt or heavy metals. Studies focusing on a combination of different stress conditions will help in understanding how plants react to these stress combinations and how possible tolerance strategies may be developed (Mittler, 2006). As heat stress often occurs in combination with other stresses the purpose of this study was to investigate the response of selected *ALDH* genes firstly to heat and secondly to a combination of heat and other abiotic stresses. To investigate the involvement of *ALDH* genes in heat stress responses ALDH mutant lines were compared with wild-type plants under heat stress regimes. The physiology of the plants was analysed including survival rate, photosynthesis, lipid peroxidation, and chlorophyll content.

## Materials and methods

### Plant materials and growth conditions

Arabidopsis of the accession Columbia (*Col-0*) was used as wild-type in this work. All transgenic plants were established in the Arabidopsis ecotype *Col-0* (Supplementary Fig. S1 at *JXB* online). The T-DNA double knock-out mutant lines *KO6/62* and *KO6/76* were made by crossing single knock-out lines (Stiti *et al.*, 2011). Transgenic plants were selected on MS agar plates containing 50 mg l^−1^ kanamycin. All plants were grown under approximately 120–150 μE m^−2^ s^−1^ light at 22 °C with a day/night cycle of 8/16 h (short day) if not otherwise stated. To induce flowering, 4-week-old plants were moved to a growth chamber with a 16/8 h photoperiod (long day). For plant growth under sterile conditions, seeds were surface sterilized in 70% (v/v) ethanol for 2 min, then in 7% (v/v) NaClO (Carl Roth; Karlsruhe, Germany) containing 0.1% (w/v) SDS for 10 min and finally seeds were rinsed four times with sterile distilled water and sown on MS-agar plates.

### Heat stress treatments

**Fig. 1.**
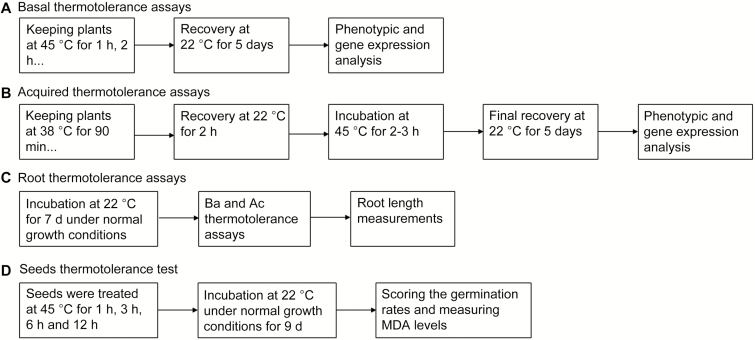
Summary of the heat treatment procedures. (A) Basal thermotolerance (Ba) was determined by keeping plants at 45 °C for 1, 2, 3, 6, 12, 24, and 72 h. After the heat treatments plants, were placed in a growth chamber at 22 °C for 5 d of recovery. (B) Acquired thermotolerance (Ac) tests were performed by keeping the plants initially for 90 min at 38 °C, then placing them at 22 °C for 2 h, before exposing them to 45 °C for 2–3 h. Final recovery was for 5 d. (C) For root growth assays plants were grown for 7 d under normal growth conditions, then the seedlings were exposed to Ba or Ac heat treatment and after that returned for 3 d to the growth chamber for root length measurements. (D) For the seed thermotolerance test, seeds were treated at 45 °C for 1, 3, 6, and 12 h and then allowed to grow for 9 d for germination analysis.

Different heat stress regimes were used. Basal thermotolerance (Ba), acquired thermotolerance (Ac), and seed thermotolerance were assayed according to the diagram in Fig. 1. All heat treatments were performed in the dark and the plants were allowed to recover in a growth chamber at 22 °C under short daylight conditions as described earlier (Larkindale *et al.*, 2005). The relative humidity in the heat stress incubator was around 70%.

Three plates of 50 seedlings each were used in a single experiment. For the experiments with mature plants, two to three 4-week-old soil-grown plants were used per pot. The plants were initially grown in short daylight conditions before they were used for the experiments. For the experiments with seeds, three plates of 50 seeds each were used in each experiment. For root growth assays, seeds were allowed to germinate and grow for 7 d under short day conditions before they were used for the experiment. All experiments were repeated three times. The control plants were kept at 22 °C in the same conditions as the heat-treated plants.

### Individual and combinatory stress treatments

We assayed different combinations of stress treatments including dehydration (D), salinity (S), high temperature (H), and wounding (W) on Arabidopsis wild-type plants, and then the plant performance was compared with that of plants subjected to a single stress treatment; the scheme of the treatments is presented in Fig. 10A. Four-week-old, soil-grown plants were subjected to different stress treatments. Plants were wounded by treating the leaf surfaces with abrasive sandpaper and afterwards kept for 4 h under short day conditions. Dehydration was imposed by withdrawing watering for 6–7 d to reach a relative water content of about 75% relative water content in the plants. Salt stress treatment was performed by watering soil-grown plants every 2 d with water containing 300 mM NaCl for 10–14 d; control plants were watered with water only.

A combination of dehydration and heat stress was performed either by subjecting dehydrated plants (typically 6–7 d) to a heat stress treatment (45 °C for 6 h) or by exposing plants first to 45 °C for 6 h followed by dehydration. A combination of wounding and heat stress was applied by wounding the leaves first followed by 45 °C for 6 h and *vice versa*. A combination of salt and heat treatment was performed by first watering the plants with 300 mM NaCl for 10–14 d followed by exposing plants for 6 h to 45 °C and *vice versa* (Fig. 10A depicts the different treatments). All experiments were performed in triplicate and repeated at least three times. Tissues were collected after the stress treatments, divided and used in parallel for molecular and metabolic analyses.

### RNA isolation and reverse transcription–PCR

Total RNA extraction was performed according to Valenzuela-Avendaño *et al.* (2005). For reverse transcription (RT)–PCR analyses, 2 μg of total RNAs was treated with 10 U of RNase-free DNase I (Roche, Mannheim, Germany) in a 10 μl reaction containing 1×DNase I buffer (20 mM Tris–HCl pH 8.4, 50 mM KCl, and 2 mM MgCl_2_) at 37 °C for 30 min. Then, 1 μl of 25 mM EDTA was added and the reaction was heated at 65 °C for 15 min to deactivate the DNase I. First-strand cDNA synthesis was performed using the Revert Aid^TM^ H Minus First Strand cDNA synthesis kit (Fermentas, Burlington, Canada).

Gene-specific primers used to amplify the first strand cDNAs are summarized in Table 1. Transcripts of the Arabidopsis *ACTIN2* (At3g18780) gene were used as reference (An *et al.*, 1996).

**Table 1.** List of primers used in this research

Gene	Name	5′–3′ sequence
*Actin2*	Ath_Actin 2_fwd	GGAATCCACGAGACAACCTATAAC
	Ath_Actin2_rev	AGGAATCGTTCACAGAAAATGTTTC
*ALDH7B4*	ALDH7B4RT-Fwd	GAAGCAATAGCCAAAGACACACGC
	ALDH7B4RT-Rev	GATATCTCGATTATCGTAGGCTCC
*ALDH3F1*	ATH-ALDH5 FWD	GAAGCCATGGAAGCTATGAAGGAGAC
	ATH-ALDH5 REV	GTCTCTGTCTCTCACTTTCCCCCTT
*ALDH3H1*	Ath-ALDH2-sense	ATCGGCGGA AGCGAGTAATTTGGTG
	Ath-ALDH2-anti	TATGGCGGATACCTGACGGCTGAATC
*ALDH3I1*	AtALDH3I1_For	GATGCAGGAAGAGATATTTGGAC
	AtALDH3I1_Rev	CATGAGTCTTTAGAGAACCCAAAG
*ALDH10A8*	AY093071-RT-fwd	GATCTTGCATGGTGGTTCCCGA
	AY093071-RT-rev	AAGCACAAAGATTTGAACAGACAGC
*ALDH10A9*	AF370333-RT-fwd	TGTTCTTTGTGGAGGAGTTCGTC
	AF370333-RT-rev	GAAGGGTCTCTTGCTTTATTGGT
*RD29B*	RD29B FOR	ACCAGAACTATCTCGTCCCAAA
	RD29B REV	CGGAGAGAGGTAGCTTTGTCAT
*HSP70*	HSP70_FOR	GGTGGTGGTACTTTTGATG
	HSP70_REV	TTGTCTTTCAGAAGATCTAT

### Protein blots

ALDH proteins were analysed using antibodies against ALDH7B4, ALDH3I1, ALDH3H1, and ALDH3F1 proteins (no antibodies were available against ALDH10A8 and ALDH10A9). Seedlings and leaves of 4-week-old plants were homogenized in Laemmli-sample buffer [62.5 mM Tris–HCl, pH 6.8, 10% (v/v) glycerol, 2% (w/v) SDS, 0.1 M DTT, 0.005% (w/v) bromophenol blue]. The extract was heated at 95 °C for 5 min. After centrifugation at 14 000 *g* for 5 min, the protein concentration of the supernatant was determined and 2 μg proteins of the supernatant was separated by 12% (w/v) SDS-polyacrylamide gel electrophoresis (Laemmli, 1970). Separated proteins were transferred onto a nitrocellulose Protran BA-85 membrane (Whatman) using a pre-chilled transfer buffer in an electro-blotting system at 70 V for 1–2 h (Towbin *et al.*, 1979). After staining the membrane with Ponceau-red solution it was kept in blocking solution [1× TBS, 0.1% (v/v) Tween-20 and 4% (w/v) skimmed milk powder] for 1 h at room temperature. ALDH proteins were analysed using antibodies against ALDH7B4, ALDH3I1, ALDH3H1, and ALDH3F1 proteins (no antibodies were available against ALDH10A8 and ALDH10A9). Anti-ALDH7B4, anti-ALDH3I1, and anti-ALDH3H1 antiserum were used for protein detection at a dilution of 1:5000 (Kotchoni *et al.*, 2006). The anti-ALDH3F1 antiserum (M. Mertens and D. Bartels unpublished) was diluted 1:1000 in blocking solution. The presence of the target protein was detected by using the ECL-Plus Western Blotting detection Kit (GE Healthcare, Braunschweig, Germany). The antigen–antibody complex was detected on the membrane by chemiluminescence under a CCD camera (Intelligent Dark Box II, Fujifilm Corp.).

### Analysis of photosynthesis parameters

Leaves of 4-week-old Arabidopsis wild-type plants and two *ALDH* double knock out mutant lines, *KO6/62* and *KO6/76*, were used to measure photosynthesis with the GFS-3000 gas exchange system (Walz, Effeltrich, Germany). Measurements were performed at 22 °C and a relative humidity of 60%. The light-saturated photosynthetic rate was determined at 0–1800 μmol m^−2^ s^−1^ with [CO_2_]=350 ppm. To generate a light response curve, photosynthetic measurements were conducted at PPFD intensities of 1800, 1500, 1000, 500, 400, 300, 200, 100, and 0. The following parameters were assessed: (i) the assimilation as yield of CO_2_, (ii) the effective quantum yield of PSII [∆*F*/*F*_m_′=(*F*_m_′−*F*_s_)/*F*_m_′], (iii) the non-photochemical quenching coefficient [NPQ=(*F*_m_−*F*_m_′)/*F*_m_′], and (iv) the maximum quantum yield of photosystem II (PSII) [*F*_v_/*F*_m_=(*F*_m_−*F*_o_)/*F*_m_].

### Lipid peroxidation assay

The amounts of lipid peroxidation products were measured in plant tissues by using the thiobarbituric acid (TBA) test, which determines the amount of malondialdehyde (MDA) as the end product of the lipid peroxidation process (Hodges *et al.*, 1999). Absorbances were read with a spectrophotometer at 440 nm (sugar absorbance), 532 nm (maximum absorbance of pinkish-red chromogen, a product of the reaction of MDA with TBA) and 600 nm (turbidity); 0.1% (w/v) TCA was used as the reference solution. The MDA contents were calculated by the formula:

$$\text{MDA equivalents }({\text{nmol ml}}^{-\text{1}})=\left[\left(A–B\right)/\text{157 }000\right]\times \text{1}0^{\text{6}}$$

where *A*=[(Abs 532_RSII_–Abs 600_RSII_)] and *B*=[(Abs 440_RSII_–Abs 600_RSII_) × 0.0571], and Abs is absorbance using reagent solution II (RSII).

$$\text{MDA equivalents }({\text{nmol g}}^{-\text{1}}\text{FW})=\text{MDA equivalents }({\text{nmol ml}}^{-\text{1}})\times \text{total volume of the extracts }\left(\text{ml}\right)/\text{g FW or number of seedlings}.$$

Reagent solution I (RSI) is 20% (w/v) TCA and 0.01% butylated hydroxytoluene (BHT), and reagent solution II (RSII) is RSI and 0.65% (w/v) 2-thiobarbituric acid (TBA).

### Determination of chlorophyll content

Leaf tissues (20–60 mg) were ground in Eppendorf tubes with metal beads under liquid nitrogen and homogenized in 2 ml 80% (v/v) aqueous acetone. The suspensions were incubated in the dark at room temperature on a shaking platform for 30 min, then centrifuged for 5 min at 12 000 *g* at room temperature (Arnon, 1949). The absorption of the extracts was measured at 663 and 645 nm. The chlorophyll content was determined by the formula:

$$\text{C }\left({\text{mg g}}^{-\text{1}}\text{FW}\right)=0.00\text{2}\times \left(\text{2}0.\text{2}\times {\text{OD}}_{\text{645}}+\text{8}.0\text{2}\times {\text{OD}}_{\text{663}}\right)/\text{g FW},$$

where C expresses the total chlorophyll content (chlorophyll *a*+chlorophyll *b*).

## Results

### Expression of *ALDH* genes in Arabidopsis plants subjected to high temperature stress

**Fig. 2.**
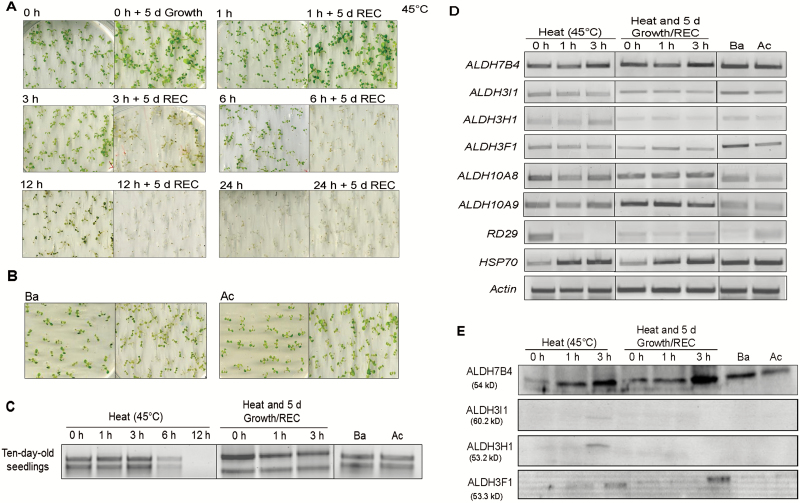
Assessment of heat tolerance and expression of *ALDH* genes in 10-day-old Arabidopsis wild-type seedlings. (A) Seedlings were subjected to 45 °C heat treatment for 0, 3, 6, 12, and 24 h without or with recovery (REC). (B) Seedlings were subjected to a basal heat stress regime (Ba) and to an acquired thermotolerance regime (Ac). REC: recovery (for details see ‘Materials and methods’). (C, D) RNA isolated from 10-day-old seedlings exposed to 0, 1, 3, 6, or 12 h to 45 °C without recovery or followed by 5 d recovery and samples subjected to a Ba or Ac treatment. (C) rRNA bands in total RNAs. (D) *ALDH*, *RD29*, or *HSP70* transcripts amplified by RT-PCR. (E) Protein blots of the samples analysed in (C). Equal loading of proteins was monitored by staining the membrane with Ponceau S (data not shown).

To assess the impact of high temperature stress on phenotypic changes of Arabidopsis plants, first the morphological changes were examined. Seedlings were exposed to 45 °C for 0–24 h and mature plants for 0–72 h. All seedlings survived 1 h heat treatment but the survival rate decreased when the treatment was increased to 3 h. Most of the seedlings showed morphological changes including brown color and wilting during the recovery period. The margins of the cotyledons showed dark green and yellowish brown color after 6 h at 45 °C and after 12 h treatment all seedlings turned yellow, bleached and did not recover (Fig. 2A).

In our conditions, the seedlings were less affected by the treatment of acquired thermotolerance than by that of the basal thermotolerance (Fig. 2B). Moreover, total RNAs from 6 and 12 h heat-treated seedlings were degraded (Fig. 2C). Based on these observations, we selected 0, 1, and 3 h exposure to 45 °C, Ba, and Ac treatments to examine the expression pattern of six stress-related *ALDH* genes (*ALDH7B4*, *ALDH3I1*, *ALDH3H1*, *ALDH3F1*, *ALDH10A8*, and *ALDH10A9*) in 10-day-old seedlings. *RD29B*, which encodes a protein that is induced in response to water deprivation but not to heat stress (Yamaguchi-Shinozaki and Shinozaki, 1994; Uno *et al.*, 2000), and *HSP70*, which encodes a heat shock protein (Mosser *et al.*, 1997; Sung *et al.*, 2001), were used as negative and positive controls, respectively. In 3 h heat-stressed seedlings *ALDH7B4* is slightly increased (Fig. 2D)*. ALDH3I1*, *ALDH3F1*, *ALDH10A8* and *ALDH10A9* transcript levels did not change under heat stress and recovery conditions, but *ALDH3H1* transcripts accumulated at a lower level during recovery. *ALDH3I1* and *ALDH3F1* transcripts accumulated under Ba stress. *HSP7*0 increased in response to high temperature and *RD29B* was hardly detected in heat-stressed samples (Fig. 2D and Supplementary Fig. S2).

To determine whether changes in transcript accumulation are reflected on the protein level, ALDH proteins were analysed. ALDH7B4 was induced after 3 h and Ba treatment in seedlings (Fig. 2E). ALDH3F1 also increased after 3 h of heat stress but not after Ba and Ac treatment. Heat stress caused only a slight increase in the expression of the ALDH3I1 and ALDH3H1 proteins after 3 h heat treatment (Fig. 2E).

**Fig. 3.**
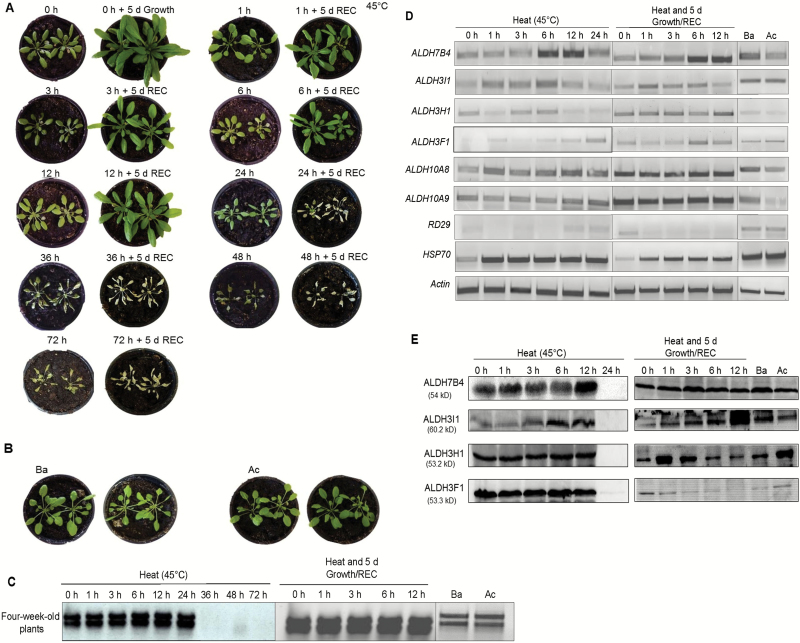
Assessment of heat tolerance and expression of *ALDH* genes in 4-week-old Arabidopsis plants. (A) Four-week-old plants were subjected to 45 °C heat treatment up to 72 h without or with recovery (REC). (B) Four-week-old plants were subjected to a basal heat stress regime (Ba) and to an acquired thermotolerance regime (Ac) (for details see ‘Materials and methods’). (C, D) RNA isolated from heat-stressed plants (B) shows the rRNA bands in total RNAs (C) and shows *ALDH*, *RD29* or *HSP70* transcripts amplified by RT-PCR (D). (E) Protein blots of the samples analysed in (C). Equal loading of proteins was monitored by staining the membrane with Ponceau S (data not shown).

When 4-week-old Arabidopsis plants were exposed to 45 °C for 1–72 h, Ba and Ac thermotolerance, no visible stress symptoms were observed up to 12 h of heat stress (Fig. 3A, B). Exposure for 24 h resulted in upwards-curled leaf margins and the entire plants were dried up after recovery. No viable plants were recovered when they were kept for 36 h or longer at 45 °C (Fig. 3A). Similarly to the seedlings, the viability of mature plants under heat stress conditions was examined based on the intactness of the extracted total RNA. Total RNAs from 36 h heat-treated 4-week-old plants were degraded (Fig. 3C). For this reason, we selected 0, 1, 3, 6, 12, and 24 h exposure to 45 °C, Ba and Ac for 4-week-old plants to analyse ALDH expression. Expression analysis of *ALDH7B4* showed that the transcripts accumulated in 6 and 12 h heat-stressed plants and declined thereafter (Fig. 3D). *ALDH3I1* transcripts increased after 1 h of heat stress, declined after 12 and 24 h of heat stress, but slightly increased after 1 h under recovery conditions. *ALDH3H1* and *ALDH3F1* transcripts are expressed at a low level in response to high temperature stress, but higher expression was observed during recovery. Transcript levels of *ALDH10A8* and *ALDH10A9* were constantly high during recovery. The levels of *ALDH7B4*, *ALDH10A8*, and *ALDH10A9* transcripts were higher under the Ba treatments than under the Ac treatment (Fig. 3D and Supplementary Fig. S3).

ALDH protein accumulation was also analysed in 4-week-old plants in response to high temperature. ALDH7B4 and ALDH3I1 were up-regulated after exposure to 45 °C after 3, 6, and 12 h of recovery conditions (Fig. 3E). ALDH3H1 and ALDH3F1 showed nearly constant expression in response to heat stress, but ALDH3F1 was reduced during recovery. The protein blots confirmed the transcript analysis and showed that mainly the *ALDH7B4* gene is up-regulated in response to high temperature.

### Examination of heat stress responses in ALDH double knock-out mutants

Plant tolerance to heat stress is determined by both the basal and acquired thermotolerance. To understand the role of the heat-inducible *ALDH* genes (*ALDH7B4* and *ALDH3I1*), the ability of Arabidopsis mutants to withstand high temperature was examined and the double mutants *KO6/62* (knock-out of *ALDH7B4* and *ALDH3I1*) and *KO6/76* (knock-out of *ALDH7B4* and *ALDH3F1*) were used.

### Survival rates

**Fig. 4.**
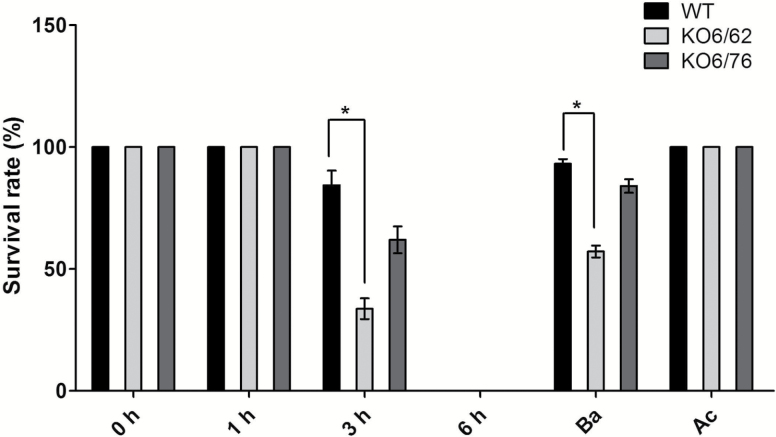
Survival of Arabidopsis wild-type and double mutant (*KO6/62* and *KO6/76)* seedlings exposed to heat stress. Percentage of 10-day-old seedlings scored as surviving after exposure to 45 °C for the different exposure times followed by 3 d growth at 22 °C (recovery), Ba and Ac thermotolerance regime. *KO6/62*: knock-out of *ALDH7B4* and *ALDH3I1*; *KO6/76*: knock-out of *ALDH7B4* and *ALDH3F1*; black bars represent wild-type plants, light-grey bars *KO6/62*, and dark grey bars *KO6/76*. All data represent means±SD (*n*=3). Asterisks indicate significant differences determined with Student’s t-test (*P<0.05).

In comparison with the wild-type, the survival rate of *KO6/62* and *KO6/76* mutants was measured in 10-day-old seedlings exposed to 45 °C for different times and then allowed to recover for 3 d. No visible difference was seen between the mutant and wild-type lines under non-stress conditions for 1 h at 45 °C and Ac treatment (Fig. 4). Wild-type seedlings grew better than the mutants after 3 h heat treatment and Ba treatment. A few mutant seedlings showed chlorosis and stopped growing after 3 h exposure to 45 °C. Wild-type plantlets showed 50% and 23% higher survival rates than the mutant lines, respectively. The survival rates of the mutant lines were also lower than wild-type under the Ba stress regime (57% *KO6/62* and 85% *KO6/76*). These results indicated that the inactivation of the *ALDH7B4*, *ALDH3I1*, and *ALDH3F1* made the Arabidopsis seedlings more sensitive to high temperature stress.

### Root length

**Fig. 5.**
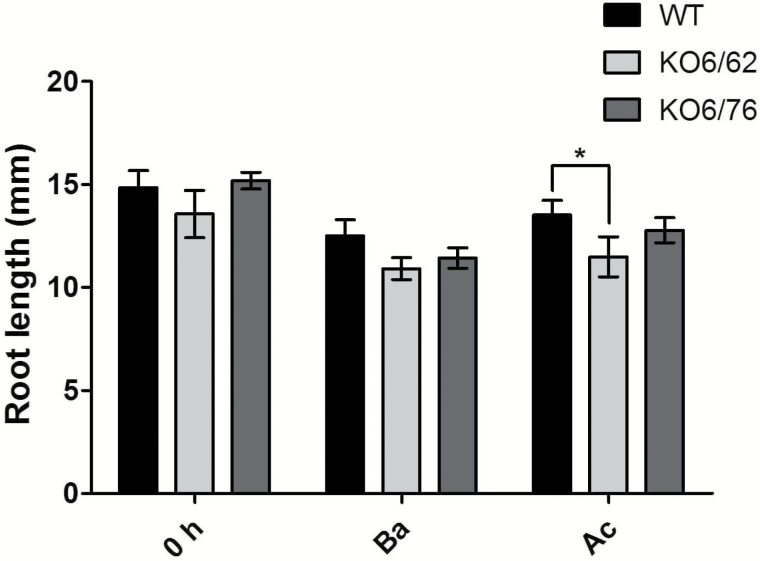
Root length of wild-type and *KO6/62* and *KO6/76* seedlings exposed to basal heat stress and acquired thermotolerance regimes. *KO6/62*: knock-out of *ALDH7B4* and *ALDH3I1*; *KO6/76*: knock-out of *ALDH7B4* and *ALDH3F1*; black bars represent wild-type plants, light-grey bars *KO6/62*, and dark grey bars *KO6/76*. The root length value was calculated as the mean±SD of 50 seedlings for each line. Asterisks indicate significant differences determined with Student’s *t*-test (*P<0.05).

Seedlings were grown on MS medium for 7 d before being subjected to Ba and Ac thermotolerance regimes. The roots of the mutant seedlings (*KO6/62* and *KO6/76*) were slightly shorter than the roots of wild-type seedlings under Ba and Ac regimes (Fig. 5). Seedlings of all genotypes were better adapted in Ac treatment than in Ba treatment.

### Lipid peroxidation assay

**Fig. 6.**
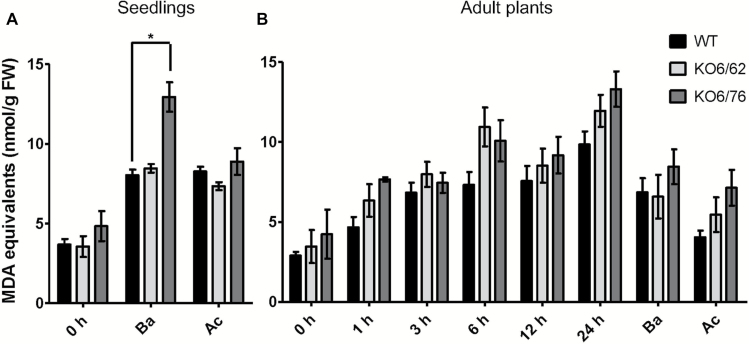
MDA levels in wild-type Arabidopsis and *ALDH* mutant plants after heat treatments. (A) Lipid peroxidation in 10-day-old wild-type, *KO6/62* and *KO6/76* mutant seedlings after basal heat stress (Ba) and acquired thermotolerance (Ac). (B) Lipid peroxidation in 4-week-old wild-type, *KO6/62* and *KO6/76* mutant plants after heat stress at different time points (1, 3, 6, 12, and 24 h exposure to 45 °C). *KO6/62*: knock-out of *ALDH7B4* and *ALDH3I1*; *KO6/76*: knock-out of *ALDH7B4* and *ALDH3F1*; black bars represent wild-type plants, light-grey bars *KO6/62*, and dark grey bars *KO6/76*. All data represent means±SD (*n*=3). Asterisks indicate significant differences determined with Student’s *t*-test (*P<0.05).

Under stress conditions, plant membrane lipids are oxidized, which often leads to the accumulation of the toxic compound MDA, which is commonly used as a marker of lipid peroxidation. Heat-shock conditions are known to cause membrane peroxidation (Wahid, 2007), and as a consequence, MDA levels could rise in heat-stressed tissues. Ten-day-old seedlings of wild-type plants, *KO6/76*, and *KO6/62* lines were subjected to the Ba and Ac heat stress. MDA levels increased upon heat stress, especially under basal heat stress. *KO6/76* and *KO6/62* mutants accumulated more MDA than the wild-type (Fig. 6A). MDA levels increased linearly with the exposure time to high temperature (45 °C) and 4-week-old *KO6/76* and *KO6/62* plants accumulated more MDA than wild-type plants under these conditions (Fig. 6B). A sharp increase of MDA was seen in double mutant lines after 6 h of heat stress, and MDA continued to accumulate up to 24 h heat treatment. MDA levels were lower under Ac treatment than under Ba treatment.

### Chlorophyll content

**Fig. 7.**
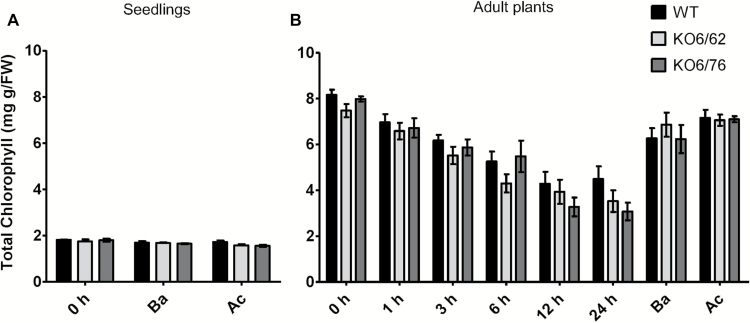
Chlorophyll content in *KO6/62*, *KO6/76*, and wild-type plants after heat stress treatment. (A) Chlorophyll content of 10-day-old wild-type and *KO6/62* and *KO6/76* mutant seedlings after basal heat stress (Ba) and acquired thermotolerance (Ac). (B) Chlorophyll content of 4-week-old wild-type, *KO6/62* and *KO6/76* mutant leaves after heat stress treatments at different time points (1, 3, 6, 12, and 24 h exposure to 45 °C). *KO6/62*: knock-out of *ALDH7B4* and *ALDH3I1*; *KO6/76*: knock-out of *ALDH7B4* and *ALDH3F1*; black bars represent wild-type plants, light-grey bars *KO6/62*, and dark grey bars *KO6/76*. All data represent means±SD (*n*=3).

Chlorophyll content was quantified as an indicator of the intactness of the photosynthetic apparatus in seedlings and 4-week-old plants. The mutated *ALDH* genes did not significantly affect the chlorophyll content in the three genotypes. However, the chlorophyll content gradually declined in wild-type and mutants with an increasing exposure time at 45 °C (Fig. 7).

### Photosynthetic efficiency

**Fig. 8.**
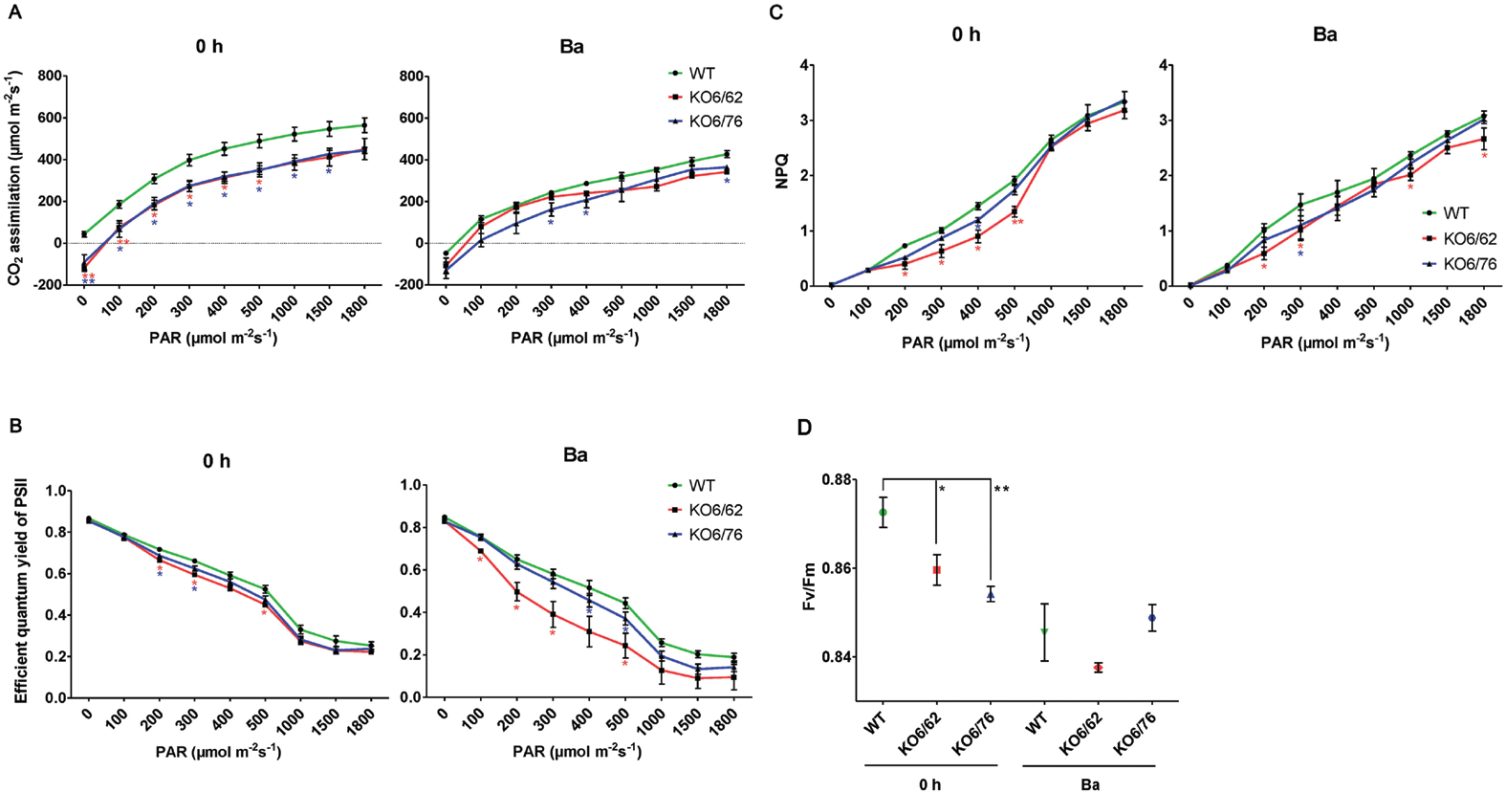
Analysis of photosynthetic parameters in wild-type and *ALDH* mutant plants *KO6/62* and *KO6/76*. CO_2_ assimilation at different light intensities (A), quantum yield of PSII (B), non-photochemical quenching (C) and kinetics of the *F*_v_/*F*_m_ in excised leaves (D) were determined in 4-week-old wild-type, *KO6/62* and *KO6/76* mutant plants under non-stress conditions and after subjection to the basal heat stress regime (Ba). Values are means±SD (*n*=8). Asterisks indicate significant differences determined with Student’s *t*-test (*P<0.05, **P<0.01).

To investigate whether *ALDH* gene expression contributes to protection of photosynthesis under high temperature conditions, photosynthetic efficiency was analysed in leaves of wild-type, *KO6/62* and *KO6/76* mutant lines under non-stress and Ba heat stress conditions (Fig. 8). The rate of CO_2_ assimilation, efficient quantum yield of PSII, non-photochemical quenching (NPQ) and maximum quantum yield of PSII (*F*_v_/*F*_m_) were determined after exposure of leaves to light of different intensities for 3 min. Ba heat treatment led to a decrease of CO_2_ assimilation in all lines (Fig. 8A). The quantum yield *q*_E_ was more reduced in *KO6/62* and *KO6/76* mutants than in wild-type after the Ba heat stress treatment (Fig. 8B). The *KO6/62* mutants showed the lowest quantum yield of all lines analysed indicating an impaired light utilization. Non-photochemical quenching (NPQ) consists of the rapid dissipation of excess excitation energy as heat. NPQ was only slightly affected in the mutants under non-stress conditions. A low NPQ activation in *ALDH* mutant lines was observed under Ba heat stress (Fig. 8C) suggesting that the capacity of dissipating excitation energy as heat was higher in wild-type than in the mutant lines. The ratio of the fluorescence *F*_v_/*F*_m_ is a measure of the maximum quantum efficiency of PSII. A reduction in *F*_v_/*F*_m_ was observed in mutant lines under control and heat stress conditions, implying a defect in energy transfer within PSII (Fig. 8D).

### Seed thermotolerance

**Fig. 9.**
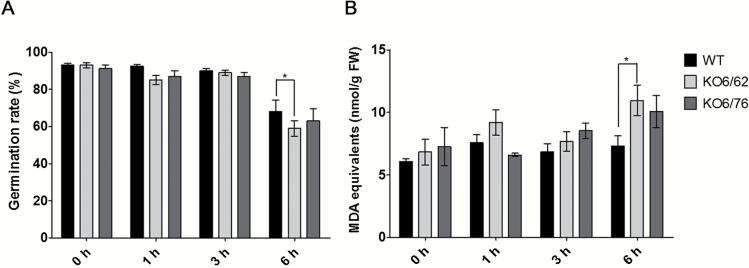
Germination and MDA content of Arabidopsis wild-type and *ALDH* mutant lines exposed to heat stress. (A) Seeds were kept for the indicated times at 45 °C and then placed on MS agar plates for germination at 22 °C. The number of germinated seeds (protruded radicals) was scored after 9 d and expressed as percentage germinated seeds. (B) MDA levels were determined in the heat-treated seeds 10 d after being placed on MS plates. *KO6/62*: knock-out of *ALDH7B4* and *ALDH3I1*; *KO6/76*: knock-out of *ALDH7B4* and *ALDH3F1*; black bars represent wild-type plants, light-grey bars *KO6/62*, and dark grey bars *KO6/76*. All data represent means±SD (*n*=3). Asterisks indicate significant differences determined with Student’s *t*-test (*P<0.05).

Some *ALDH* genes are abundantly expressed in embryos of seeds (Kirch *et al.*, 2005; Stiti *et al.*, 2011; Missihoun *et al.*, 2014), and therefore we investigated whether *ALDH* genes are involved in seed thermotolerance. The seeds of the *ALDH* knock-out mutants *KO6/62* and *KO6/76* were placed on MS medium to test their ability to germinate or recover within 9 d at 22 °C after being kept at 45 °C for 0, 1, 3, and 6 h (Fig. 9A). Nearly 100% germination was observed for wild-type, *KO6/62* and *KO6/76* under non-stress conditions (22 °C), but the germination ability was reduced in the *KO6/62* and *KO6/76* mutants compared with wild-type seeds after 1, 3, or 6 h exposure to 45 °C. To test whether the loss of germination was due to lipid peroxidation, MDA levels were determined in the heat-treated seeds 10 d after being placed on MS plates (Fig. 9B). Mutant seeds exposed to 45 °C for 3 and 6 h accumulated more MDA than the wild-type seeds. These results demonstrate that loss of *ALDH* genes affects the ability to germinate at high temperature.

### Phenotype analysis and expression of *ALDH* genes in Arabidopsis plants subjected to a combination of dehydration, wounding, salt, and/or heat stress

**Fig. 10.**
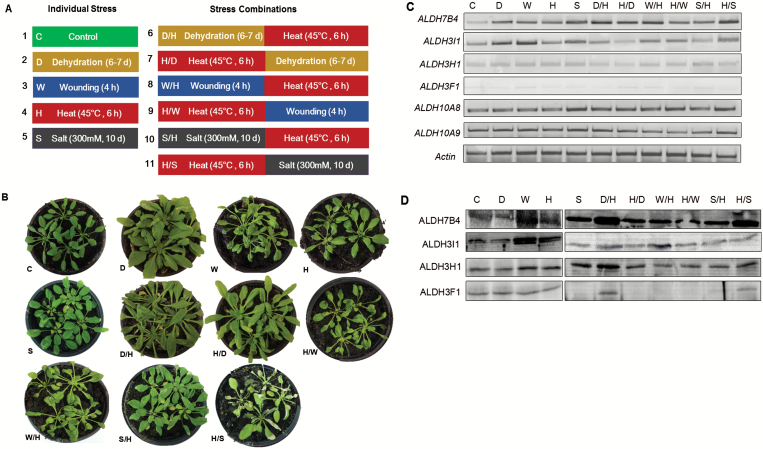
Stress combinations studied in 4-week-old Arabidopsis plants. (A) The experimental design of stress applications. C, control; D, dehydration; W, wounding; H, heat; S, salt; D/H, dehydration followed by heat stress; H/D, heat stress followed by dehydration; W/H, wounding stress followed by heat stress; H/W, heat stress followed by wounding; S/H, salt stress followed by heat stress; H/S, heat stress followed by salt stress. (B) Phenotype analysis of 4-week-old Arabidopsis plants subjected to a single stress and stress combinations. (C) Transcript accumulation analysed by RT-PCR using gene specific primers to amplify the transcripts from total RNA (2 μg) extracted after different stress treatments (for details see ‘Materials and methods’). (D) Protein blot analysis of protein extracts from 4-week-old plants subjected to different stress treatments (C).

We have shown in our previous studies that stress-related *ALDH* genes are involved in tolerance to different abiotic stress factors when applied individually in the laboratory (Sunkar *et al.*, 2003; Kotchoni *et al.*, 2006; Stiti *et al.*, 2011). In this study, we investigated how the stress-related *ALDH* genes are expressed in response to multiple stresses (Fig. 10A). Plants that were subjected to wounding stress (W), dehydration–heat stress (D/H), heat–wounding (H/W), heat–salt (H/S) stress and wounding–heat (W/H) showed clear signs of injuries, and some leaves after D/H and H/S treatment turned yellow and dried up (Fig. 10B). However, plants subjected to heat–dehydration (H/D) stresses did not show severe symptoms, probably because those plants recovered from the heat stress during 7 d of dehydration that followed at a non-stress temperature (22 °C). For each stress combination the transcript and protein levels of *ALDH7B4*, *ALDH3H1*, *ALDH3I1*, *ALDH3F1* and two betaine aldehyde dehydrogenase genes *ALDH10A8* and *ALDH10A9* were determined as in the previous sections (Fig. 10C). RT-PCR analysis indicated that *ALDH7B4* transcripts increased under all single stresses applied and accumulated to even higher levels after stress combinations D/H, H/D, W/H and H/S. Expression of *ALDH3I1* was induced upon dehydration, wounding and slightly in response to salt stress, but transcripts remained low after a combination of stress treatments. *ALDH3H1* and *ALDH3F1* accumulated at a low level in nearly all treatments. Expression of *ALDH10A8* and *ALDH10A9* genes was constant and did not change in response to either single stresses or stress combinations. Protein blots showed that the ALDH7B4 protein was induced by all stress conditions, and accumulated abundantly under W, D/H, and H/S stress treatments (Fig. 10D). ALDH3I1 protein was up-regulated in response to W and H treatment and accumulated slightly after D/H and W/H. The ALDH3H1 protein level increased under W, S, D/H, and H/D, but to a lesser extent under heat and H/S treatments. The ALDH3F1 protein level did not change in response to individual stresses and became detectable only in response to D/H and H/S stress combinations (Fig. 10D). These results indicated that the transcripts and proteins of ALDH7B4 and ALDH3H1 were the most highly induced of the *ALDH* genes tested under D/H and H/S stress combinations.

### ALDH double knock-out mutants exposed to stress combinations: malondialdehyde and chlorophyll contents

**Fig. 11.**
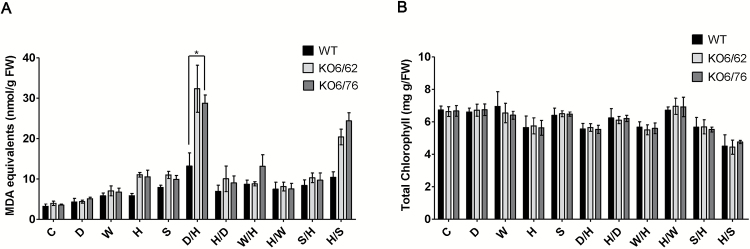
MDA and chlorophyll levels in response to single stresses and a combination of stresses. MDA content (A) and chlorophyll levels (B) were determined in 4-week-old wild-type and *KO6/62* as well as *KO6/76* mutant lines. C, control; D, dehydration; W, wounding; H, heat; S, salt; D/H, dehydration followed by heat stress; H/D, heat stress followed by dehydration; W/H, wounding stress followed by heat stress; H/W, heat stress followed by wounding; S/H, salt stress followed by heat stress; H/S, heat stress followed by salt stress. Black bars represent wild-type plants, light-grey bars *KO6/62*, and dark grey bars *KO6/76*. All data represent means±SD (*n*=3). Asterisks indicate significant differences determined with Student’s *t*-test (*P<0.05).

To further analyse the role of stress-related ALDHs in the plant tolerance to heat, we compared the MDA and chlorophyll levels in wild-type and *KO6/62* as well as *KO6/76* mutant lines subjected to a combination of heat with other abiotic factors. In comparison with other stress conditions, D/H and H/S caused a significant increase in accumulation of MDA, and mutant lines accumulated significantly higher MDA levels than stressed wild-type plants under these conditions (Fig. 11A). These results corroborate a role for *ALDH* genes in stress protection by lowering lipid peroxidation. The leaves from heat, D/H, W/H, S/H and H/S treatments have lower chlorophyll contents than under other conditions; however, the chlorophyll levels decreased in the wild-type and knock-out mutants similarly (Fig. 11B).

## Discussion

### Expression profiles of the Arabidopsis *ALDH* genes in response to heat stress

We analysed the function of selected *ALDH* genes in Arabidopsis plants subjected to heat stress either alone or in combination with dehydration, salinity, or wounding stress. The analysis of the expression of selected *ALDH* genes in response to heat stress indicated that *ALDH* genes play a crucial role in protecting plants from high temperature damage. The *ALDH* genes were shown to be involved in the aldehyde-detoxification and ROS-elimination processes. Previous studies in our lab showed that the expression of selected *ALDH* genes was induced in response to various abiotic stresses, such as dehydration, NaCl, heavy metals (Cu^2+^ and Cd^2+^), H_2_O_2_ and ABA treatment (Sunkar *et al.*, 2003; Kirch *et al.*, 2005). Overexpression of *ALDH* genes improved stress tolerance and knock-out of *ALDH* genes showed increased sensitivity to stress (Kirch *et al.*, 2001; Missihoun *et al.*, 2011, 2012). In this study, we found that the expression of *ALDH* genes, particularly *ALDH7B4*, was induced by heat stress. The *ALDH3I1* expression was weakly induced by high temperature in mature plants. The expression of *ALDH3H1* and *ALDH3F1* was weak during heat treatment but it increased during recovery (Figs 2D, E and 3D, E). Choi and Lee (2012) found that in *Antarctic microalga* an increase in total ALDH activity occurred following heat exposure. Their results indicate that ALDH 5 and ALDH 6 isozymes have important roles in heat tolerance. Hibino *et al.* (2001) showed that the mangrove betaine aldehyde dehydrogenase enzymes (BADH) were very stable at high temperature and contributed to heat tolerance. This is supported by the studies here, because the two BADH-encoding genes in Arabidopsis, *ALDH10A8* and *ALDH10A9*, are induced during recovery (Figs 2D, E and 3D, E). This suggests that *ALDH10A8* and *ALDH10A9* may function in the plant response to heat stress through the biosynthesis of compatible solutes. As early studies revealed Arabidopsis does not accumulate high amounts of glycine betaine (Missihoun *et al.*, 2015), the increase of the expression of *ALDH10A8* and *ALDH10A9* could be for the biosynthesis of γ-aminobutyric acid, which was shown to alleviate the effect of heat stress in plants (Locy *et al.*, 2000; Nayyar *et al.*, 2014).

### Double knock-out mutants of ALDH3 and ALDH7 lead to heat-sensitive plants

Heat stress often decreases root growth, the number of roots and root diameter (Khurana *et al.*, 2013). Hancock *et al.* (2014) pointed out that in potato, heat stress caused a dramatic change in allocation of assimilation products between shoot and roots and reduced the starch content and dormancy.  Tian *et al.* (2009) reported that in heat-adapted thermal *Agrostis scabra*, the architecture changes and hypocotyls and petioles elongate, resembling the morphological responses of high temperature stress. To evaluate the importance of *ALDH* genes in the plant tolerance to heat, seedlings of two double mutants of *ALDH* genes (*KO6/62* and *KO6/76*) were examined for their survival rate and root growth under heat stress in our study. Similarly to the observation by Tian *et al.* (2009), the two *ALDH* mutants showed heat-defective phenotypes, including lower survival rates and slightly shorter root length under different heat stress treatments (Figs 4 and 5). These findings suggest that *ALDH3I1*, *ALDH3F1*, and *ALDH7B4* are involved in protecting the plant in the early growth period from heat damage. Indeed, Bouché *et al.* (2003) found that T-DNA mutants of *SSADH1* (*ALDH5F1*) in Arabidopsis are dwarf and display enhanced sensitivity to heat stress in parallel with a rapid increase in hydrogen peroxide levels, suggesting that this gene restricts levels of ROS intermediates in plant defense against environmental stress. Like other abiotic stresses, heat stress results in the production of ROS and triggers oxidative stress responses (Potters *et al.*, 2007). ROS cause damage to a wide range of cellular components such as the photosynthetic apparatus, and mitochondrial and chloroplast electron transport chains (Xu *et al.*, 2006). The heat stress increased leaf temperature, which reduced the antioxidant enzyme activities and ultimately increased MDA contents, in leaves of rice and wheat plants (Rahman *et al.*, 2009; Savicka and Škute, 2010). Hence, membrane lipid saturation is considered as an important element in high-temperature tolerance. In our study, a high MDA level was found in the *ALDH* mutants compared with the wild-type plants under heat stress (Fig. 6). This indicates that *ALDH3I1*, *ALDH3F1*, and *ALDH7B4* contribute to the detoxification of ROS during heat-induced oxidative stress, which is in agreement with previous observations on the *ALDH3I1* and the *ALDH7B4* single knock-out mutants subjected to salinity and dehydration stress (Kotchoni *et al.*, 2006).

Photosynthesis is one of the most heat sensitive physiological processes in plants. Photosystem II (PSII) activity greatly decreases and even stops under heat stress (Morales *et al.*, 2003). The ability of the plant to maintain leaf gas exchange and CO_2_ assimilation rates under heat stress is directly correlated with heat tolerance (Yang *et al.*, 2006). In our study heat-treated leaves of *ALDH* mutant lines exhibited a lower CO_2_ assimilation rate, reduced efficient quantum yield, lower NPQ activation, and a reduction in *F*_v_/*F*_m_ in comparison to Arabidopsis wild-type, which suggested that the loss of *ALDH* genes affects the plant photosynthetic efficiency (Fig. 8). Similar observations were made in soybean, rice, tobacco and oak leaves (Hurkman *et al.*, 2009; Suwa *et al.*, 2010; Djanaguiraman *et al.*, 2011; Tan *et al.*, 2011), where heat stress decreased total chlorophyll content, chlorophyll *a* content, chlorophyll *a*/*b* ratio, and *F*_v_/*F*_m_ ratio under heat stress conditions. Combined with our study, this indicated that the protective role of *ALDH3I1*, *ALDH3F1*, and *ALDH7B4* is required to maintain membrane fluidity and to support leaf gas exchanges and photosynthesis. The ALDH enzymes may function by reducing lipid peroxidation of chloroplast and thylakoid membranes under high-temperature conditions.

Germination is the plant growth stage most affected by heat (Hasanuzzaman *et al.*, 2013). Toh *et al.* (2008) found that in Arabidopsis seeds reduced germination rates and plant emergence, abnormal seedlings, poor seedling vigor, and reduced radicle and plumule growth of germinating seedlings are the major effects caused by heat stress. In wheat and tomato, the germination and the early growth of seedlings were strictly prohibited under heat stress (Cheng *et al.*, 2009). Similarly, we found that seeds of the ALDH mutants exposed to high temperatures had lower germination rate than the wild-type seeds. In parallel, high amounts of MDA accumulated in both mutant lines (Fig. 9). These observations are consistent with other previous studies. In the rice seeds, OsALDH7 is involved in removing aldehydes formed by oxidative stress during seed desiccation. The OsALDH7 mutant seeds were more sensitive to an accelerated aging treatment and accumulated more MDA than the wild-type (Shin *et al.*, 2009). Our results indicate that plant ALDH enzymes play an important role in maintaining seed viability by detoxifying the aldehydes generated by lipid peroxidation. Altogether, the results of this study demonstrated that ALDH enzymes contribute to the survival of plants under high temperature.

### ALDH7B4 contribute to a combination of dehydration, salt, and/or heat stress tolerance

In the field, plants are often exposed to several stressors simultaneously. For this reason, there is a need to understand the nature of responses to multiple stresses (Suzuki *et al.*, 2014). The response of plants to a combination of different stresses is difficult to predict compared with the response of plants to individual stressors (Mittler, 2006).

Vile *et al.* (2012) have shown that Arabidopsis plants increase their stomatal conductance during heat stress in order to cool off their leaves by transpiration. However, if heat stress occurs simultaneously with drought, plants are not able to open their stomata and their leaf temperature would increase by 2–5 °C (Rizhsky *et al.*, 2002, 2004). In agreement with this, we observed that heat stress alone had a detrimental effect on Arabidopsis plants but a combination of heat stress with other stressors showed more severe growth defects (Figs 2A, 3A and 10B).

To better understand how the ALDHs are regulated under multiple stresses, we have analysed their expression patterns. We found that heat combined with dehydration resulted in an ALDH7B4 expression higher than in the case of a single stress (Fig. 10C, D). Consistently, transcriptome analysis of Arabidopsis plants subjected to a combination of heat and drought found the *ALDH7B4* transcript more than fourfold elevated (Rizhsky *et al.*, 2004). Our observation about the expression of the *ALDH7B4* gene was therefore supported by the conclusion that the combined effects of heat and drought were generally additive (Suzuki *et al.*, 2014). In parallel with the gene expression data, we found that *ALDH* knock-out plants accumulate more MDA than wild-type plants under H/D and D/H (Fig. 11). However, no significant effect was seen on the chlorophyll content in Arabidopsis plants under H/D and D/H compared with individual stresses (Fig. 11).

We noted that the order in which the stresses were applied also matters. We observed that the expression of ALDHs was higher after D/H than H/D, and consistently, the plants had more severe symptoms in the D/H assay than in the H/D assay (Fig. 10B). This suggests that dehydration alone may cause more damage to the plant than heat and that the ALDH regulon in the dehydration response may overlap but supersede the heat response.

As in the case of combined drought and heat stress, the effects of salinity stress could be exacerbated when combined with heat stress, because enhanced transpiration could result in enhanced uptake of salt (Keleş and Öncel, 2002; Wen *et al.*, 2005). Suzuki *et al.* (2016) showed that Arabidopsis plants were more susceptible to the combination of salt and heat stress than to each of the different stresses applied individually. Based on our RT-PCR and protein blot results, ALDH7B4, ALDH3I1, and ALDH3H1 are induced by either heat or salinity stress. An increased expression of ALDH7B4 was observed under H/S stress than under single stress (Fig. 10C, D). More ALDH7B4 accumulates during H/S than during S/H and a significant elevation of MDA was detected during the H/S condition. Higher accumulation of MDA in mutant plants than wild-type plants (Fig. 11). Our results are consistent with those by Suzuki *et al.* (2016). In contrast to drought, salinity, and heat, wounding stress imposes a mechanical damage to the plant by enabling a pathogen invasion. We found that the phenotype of the Arabidopsis plants caused by a combination of heat and wound stress was more severe than that observed for either individual stress treatment (Fig. 10B). Abiotic stressors were shown to weaken the defense mechanisms of plants (Mittler and Blumwald, 2010; Atkinson and Urwin, 2012), which suggests that the regulation of ALDHs by heat stress may overlap with that of drought, salinity, wounding, and pathogens. Indeed, the analysis of the *ALDH7B4* promoter activation indicated that *ALDH7B4* could be induced by pathogen and herbivore attacks as well as by drought and high salinity (Missihoun *et al.*, 2014). The *ALDH7* homolog in rice was also induced by the fungus (Wu *et al.*, 2007). When wounding and heat stress were applied in combination, besides ALDH7B4, other ALDHs were not induced in response to both H/W and W/H, although they were highly regulated by wound stress alone. ALDH7B4 proteins were more highly expressed during W/H than during H/W or H but were lower than W. The ALDH3I1 also showed a higher expression after W/H than after H/W (Fig. 10D). Similarly, virus-treated plants displayed enhanced expression of defense genes, which was abolished in plants additionally subjected to heat and drought stress (Prasch and Sonnewald, 2013). Suzuki *et al.* (2014) suggested that after stress treatment Ca^2+^ and ROS are produced independently of biotic and abiotic stress applications and ABA seems to be centrally positioned between ROS and SA signaling. We found that the plants accumulated more MDA during W/H than during single stress. The ALDH double knock-out mutants have higher MDA levels than wild-type plants under stress conditions (Fig. 11). Therefore, we proposed that ALDH7B4 may also function as peroxidation-inhibiting enzyme during a combination of heat and wounding stress.

Taken together, our results indicate that heat and stress combinations induce the expression of *ALDH* genes. The expression patterns of *ALDH* genes vary depending on the stress factors and combinations thereof. *ALDH7B4* was found to be strongly induced by D/H and H/S and slightly in W/H. ALDH knock-out mutants are more sensitive to heat and stress combinations than wild-type plants indicating that *ALDH* genes contribute to heat stress tolerance. The inactivation of *ALDH3I1*, *ALDH3F1*, and *ALDH7B4* resulted in high MDA levels within the plant during heat stress and combination with drought, high salinity and wounding stresses. These results indicate that ALDHs act as a major antioxidant defense machinery to eliminate ROS during stress combinations.

## Conclusions

Our study attempted to identify the role of Arabidopsis *ALDH* genes in response to high temperature and stress combinations (heat combined with dehydration, wounding or salinity). The data presented here provide information on the expression profiles of *ALDH* genes under heat stress and combined stress conditions. The results show that *ALDH* genes, particularly *ALDH7B4*, are induced by heat stress in seedlings as well as adult plants. An increased expression of *ALDH7B4* was also observed under heat combined with dehydration, salinity or wounding stress. Measurements of changes in physiological parameters of T-DNA double mutants of *ALDH* genes and wild-type plants demonstrated that mutant lines are more sensitive to heat stress and combined stress conditions. These results indicate that *ALDH* genes could play a role in protecting plants from high temperature damage and combined stress conditions.

## Supplementary data

Supplementary data are available at *JXB* online.

Fig. S1. Morphological phenotypes of Arabidopsis plants.

Fig. S2. Quantification of *ALDH* gene expression in 10-day-old wild-type seedlings in response to heat stress.

Fig. S3. Quantification of *ALDH* gene expression in 4-week-old wild-type plants in response to heat stress.

## Supplementary Material

Supplementary_Figures_S1_S3

## Abbreviations:

ALDH: aldehyde dehydrogenase
KO: knock-out
MDA: malondialdehyde
ROS: reactive oxygen species.
