# Supplementary material for: The role of Arabidopsis aldehyde dehydrogenase genes in response to high temperature and stress combinations
Source: J Exp Bot. 2017 Jul 11;68(15):4295–308. doi: 10.1093/jxb/erx194 (PMC5853279; doi:10.1093/jxb/erx194)

Supplementary Figures S1-S3

Fig. S1. Morphological phenotypes of *A. thaliana* plants. (A) *A. thaliana* wild-type ten-day-old seedlings under control conditions. (B) *A. thaliana* wild-type four-week-old plants under control conditions

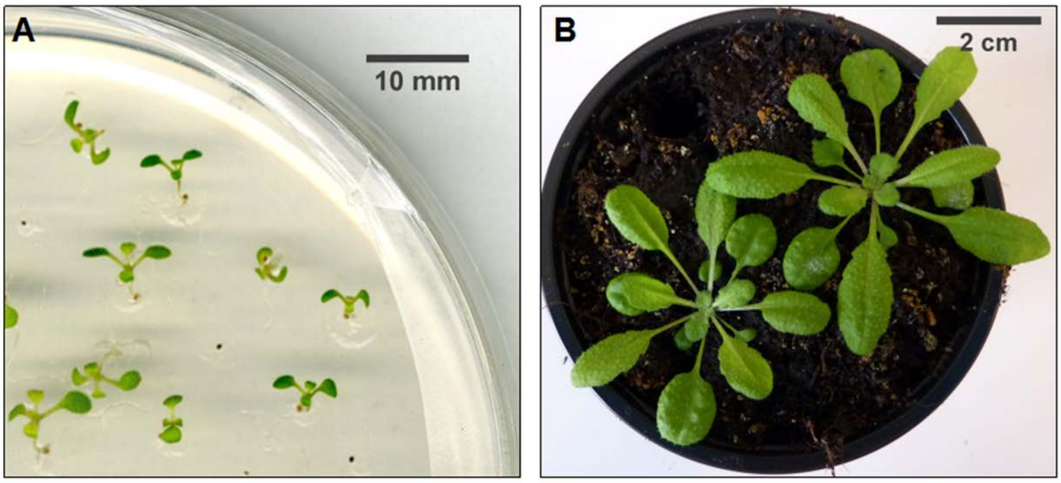

Fig. S2. Quantification of *ALDH* genes expression relative to actin transcript levels in ten-day-old wild-type seedlings of *A. thaliana* in response to heat stress.

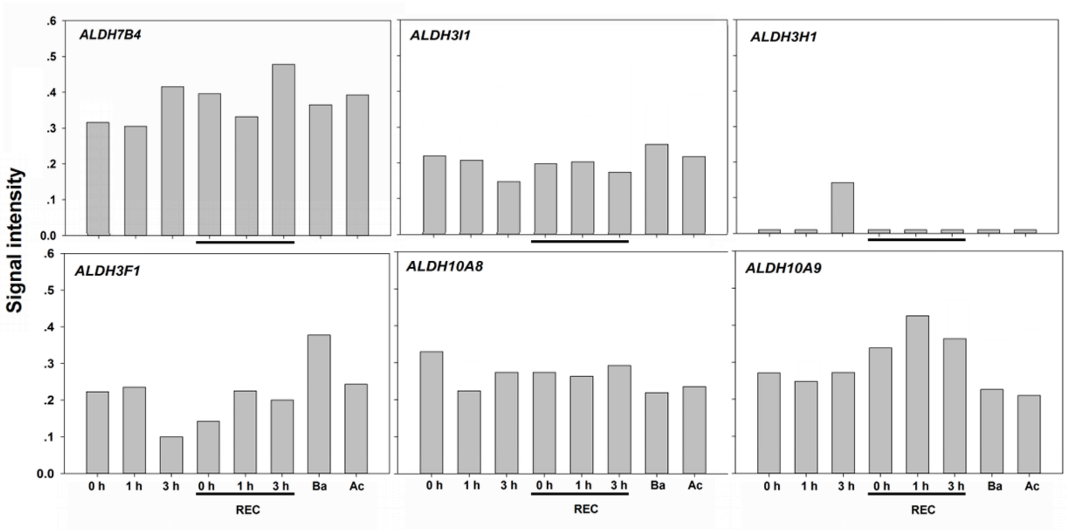

Fig. S3. Quantification of *ALDH* genes expression relative to actin transcript levels in four-week-old wild-type plants of *A. thaliana* in response to heat stress.

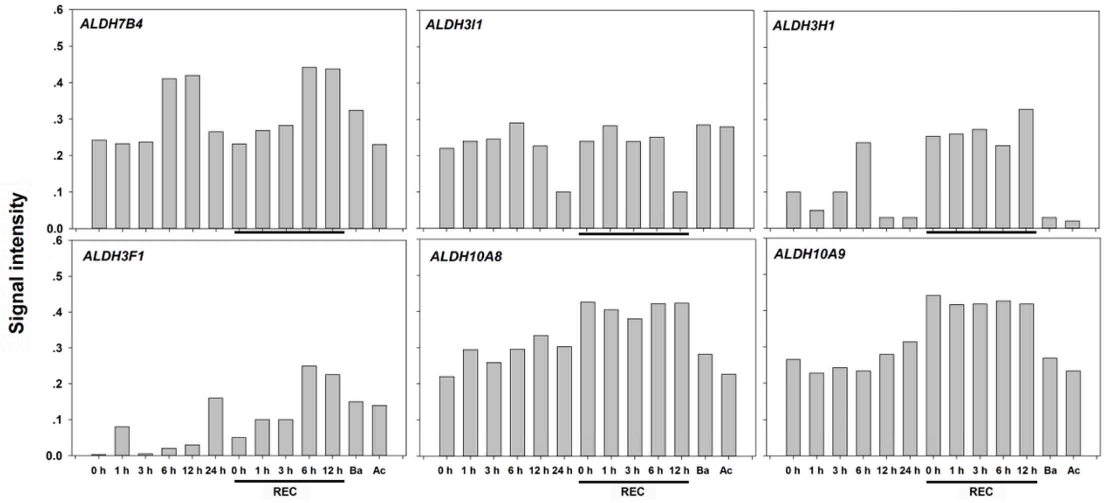

Supplement: Supplementary_Figures_S1_S3 [file erx194_suppl_supplementary_figures_s1_s3.pdf]
